# The incidence rates of endometrial hyperplasia and endometrial cancer: a four-year population-based study

**DOI:** 10.7717/peerj.2374

**Published:** 2016-08-24

**Authors:** Jin-Sung Yuk

**Affiliations:** Obstetrics and Gynecology/School of Medicine, MizMedi Hospital, Eulji University, Seoul, Republic of Korea

**Keywords:** Incidence, Endometrial hyperplasia, Endometrial cancer

## Abstract

**Introduction:**

The aim of this study was to determine the incidence rates of endometrial hyperplasia (EH) and endometrial cancer (EC) in the Republic of Korea using national insurance claim data generated from 2009 to 2012.

**Materials and Methods:**

Data that were generated from 2009 to 2012 were sourced from the Korean Health Insurance Review and Assessment Service-National Inpatients Sample database. The data from women who were assigned diagnosis codes representing EH or EC within 1 month of being assigned codes that corresponded to procedures that included endometrial biopsies and several types of gynecologic surgeries to obtain endometrial pathology samples, were selected for analysis.

**Results:**

Data from 2,477,424 women were entered into the database between 2009 and 2012, and the data from 1,868 women with EH and 868 women with EC were extracted for analysis. The mean ages of the patients were 44.1 ± 0.4 years for those with EH and 52.7 ± 0.6 years for those with EC. The EH and EC incidence rates were 37 per 100,000 woman-years and 8 per 100,000 woman-years, respectively. The EH and EC incidence rates peaked when the women were in their late forties and fifties, respectively.

**Conclusions:**

The EH and EC incidence rates determined in this study were somewhat lower than those determined from previous studies. Further studies are required that adjust the data for race, menopausal hormone therapy, and obesity.

## Introduction

Endometrial hyperplasia (EH) is characterized by the excessive growth of the endometrial glands and it can progress to endometrial cancer (EC) (Armstrong et al., 2012). Of the two systems used to classify EH, namely, the World Health Organization (WHO) and the Endometrial Intraepithelial Neoplasia (EIN) classification systems (Salman et al., 2010), the EIN system is more objective than the WHO system, but the latter is more widely used (Baak et al., 2005; Armstrong et al., 2012).

EH pathophysiology is associated with the excessive and continuous stimulation of the endometrium by estrogen (Epplein et al., 2008). The EH risk factors include chronic anovulation, polycystic ovary syndrome, obesity, tamoxifen therapy, and estrogen-only hormone therapy (Epplein et al., 2008; Carlson et al., 2012; Armstrong et al., 2012). The EH incidence is 133–208 per 100,000 woman-years, and the incidence rates of the EH subtypes are 121 per 100,000 woman-years for non-atypical EH and 16.8 per 100,000 woman-years for atypical EH (Reed et al., 2009; Lacey et al., 2012).

Previous studies analyzed data that were generated between 1980 and 2003, and menopausal hormone therapy (MHT) was actively used during this time (Reed et al., 2009; Lacey et al., 2012). Furthermore, estrogen-only hormone therapy, which is a type of MHT, is a risk factor for EH (Epplein et al., 2008; Carlson et al., 2012; Armstrong et al., 2012). The use of MHT declined globally following the Women’s Health Initiative (WHI) study that investigated and described the MHT risks in 2002 (Rossouw et al., 2002; Haas et al., 2004; Anderson et al., 2004; Guay et al., 2007). Hence, new studies are necessary to determine the EH and EC incidence rates following the WHI study, and to compare these rates with those reported before the WHI study.

The aim of this study was to determine the EH and EC incidence rates in the Republic of Korea using national insurance claim data generated between 2009 and 2012.

## Materials and Methods

### Sample

Korean national health insurance covers the provision of a broad range of medical care services to over 98% of the residents of South Korea, which has a population of about 49 million people (Kim, Kim & Kim, 2014). The Health Insurance Review Agency is a neutral entity that is positioned between the Korean National Health Insurance Service Corporation and the medical institutions, and it assesses almost all medical payments (Kim, Kim & Kim, 2014). The Korean Health Insurance Review and Assessment Service-National Inpatients Sample (HIRA-NIS) is a representative annual sample of one million patients’ data that is extracted from the entire South Korean population. A probabilistic weighted sample extraction method is used to extract the samples for the HIRA-NIS (inpatient group: 13% extraction of the total inpatient population; outpatient group: 1% extraction of the total outpatient population), and the samples are stratified according to sex and age (Kim, Kim & Kim, 2014).

Yuk et al. (2015) and Yuk et al. (2016) recently reported two studies that described the incidence of unexpected uterine malignancies in women who had undergone myomectomies: one of these studies used the HIRA-NIS, while the other study used whole claims data (Yuk et al., 2015; Yuk et al., 2016). The results from the two studies were consistent, which indicated that the HIRA-NIS represented the incidence of unexpected uterine malignancies excellently.

### Subject selection

We used the HIRA-NIS data that were generated between January 1, 2009 and December 31, 2012 (serial numbers: HIRA-NIS-2009-0066, 2010-0084, 2011-0063, and 2012-0058) (Kim, Kim & Kim, 2014). The diagnosis codes in the 10th revision of the International Classification of Diseases were used to obtain data from women who had been diagnosed with EH (N85.0: endometrial glandular hyperplasia and N85.1: endometrial adenomatous hyperplasia [atypia]) or with EC (C54.1: malignant neoplasm of the endometrium). The patients had endometrial disease if they had been assigned diagnosis codes for EH or EC within 1 month of undergoing procedures that included dilatation and curettage (procedure code: R4521), aspiration biopsies (procedure code: C8573), simple curettage (procedure code: C8574), simple abdominal hysterectomy (procedure code: R4145), complex abdominal hysterectomy (procedure code: R4146), subtotal hysterectomy (procedure code: R4130), vaginal hysterectomy (procedure code: R4202), and vaginal hysterectomy with anterior and posterior repairs (procedure code: R4203); the procedure codes were derived from health insurance medical care expense claim forms. Patients with EH diagnosis codes had been additionally assigned drug codes that included medroxyprogesterone acetate, levonorgestrel-releasing intrauterine system, and gonadotropin-releasing hormone agonists.

### Statistical analysis

R version 3.1.3 (The R Foundation for Statistical Computing, Vienna, Austria; http://www.R-project.org/) was used to manage and analyze the data. A weighted Pearson’s chi-square test was used to analyze the categorical data. Weighted analyses were used to calculate the means of the continuous data. The results were considered statistically significant when *p* was <0.05. All of the statistical tests were two tailed.

### Ethical statement

Since the HIRA-NIS dataset uses anonymous identification codes to protect patients’ personal information, institutional review board approval was not required.

## Results

**Figure 1:**
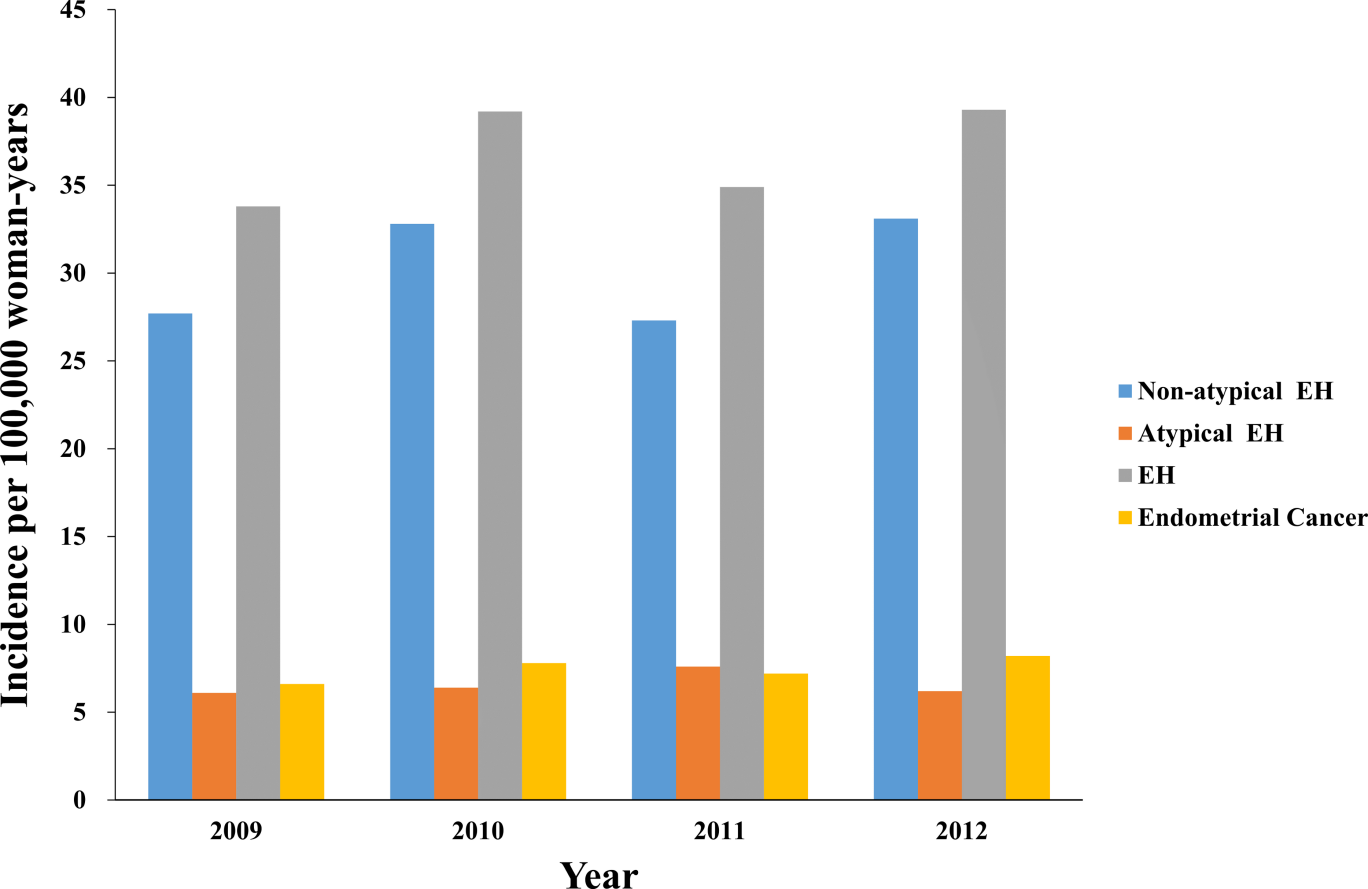
The annual trends in the endometrial hyperplasia and endometrial cancer incidence rates from 2009 to 2010. EH, endometrial hyperplasia.

**Figure 2:**
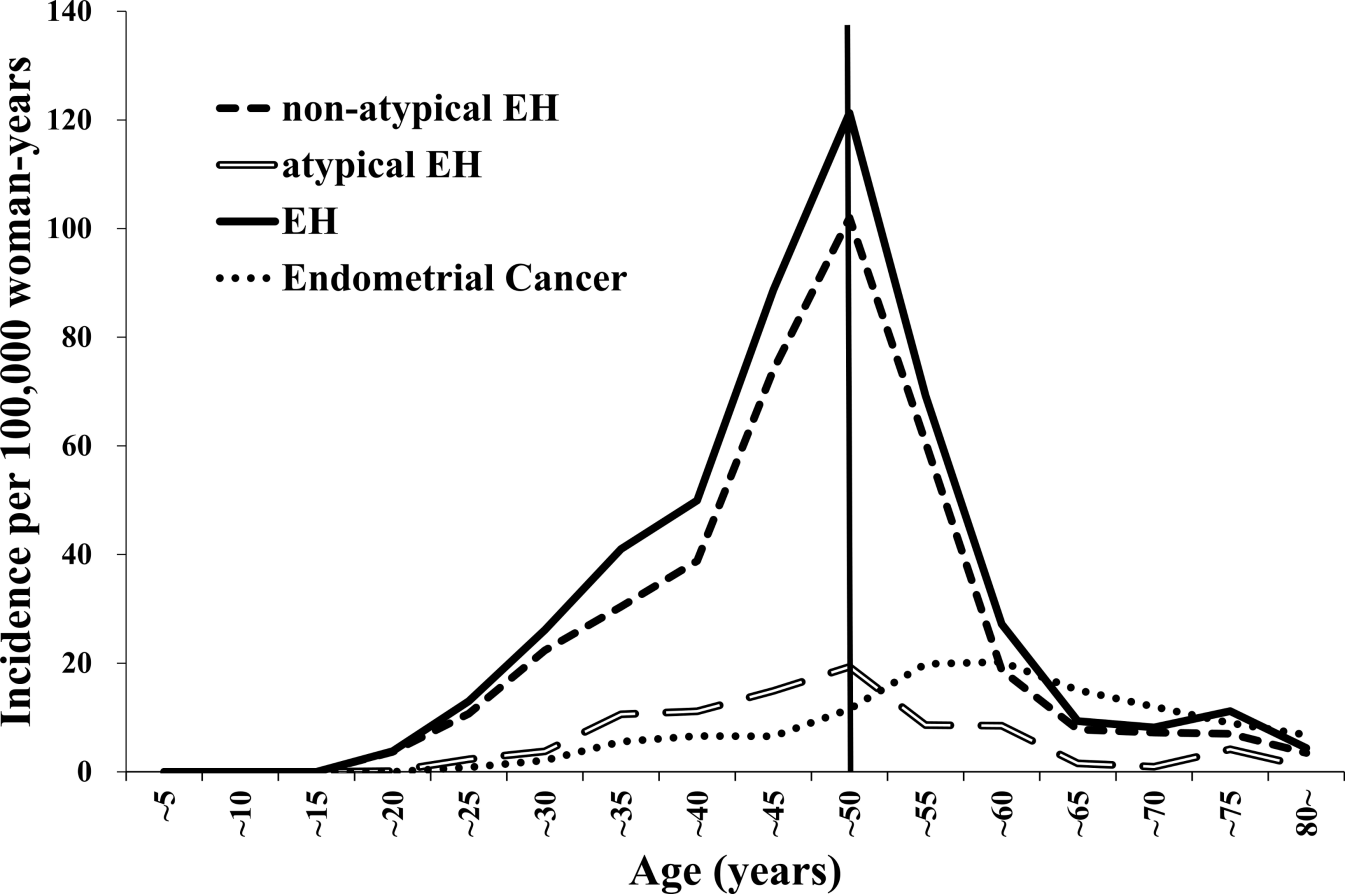
The trends in the endometrial hyperplasia and endometrial cancer incidence rates from 2009 to 2012 stratified according to 5-year age increments. EH, endometrial hyperplasia.

Data from 2,477,424 women were entered into the database from 2009 to 2012, and the data from 1,868 women with EH and 868 women with EC were extracted for analysis (Table 1). The mean ages of the patients were 44.1 ± 0.4 years and 52.7 ± 0.6 years for those with EH and EC, respectively. The incidence rates were 37 per 100,000 woman-years (95% confidence interval (CI) 34–40 per 100,000 woman-years) for EH and 7 per 100,000 woman-years (95% CI (7–8) per 100,000 woman-years) for EC. Table 1 presents the characteristics of the women with EH or EC. Figure 1 presents the annual trends in the EH and EC incidence rates. Table 2 and Fig. 2 present the EH and EC incidence rates stratified into 5-year age increments. The EH incidence, which included non-atypical EH and atypical EH, peaked when the women were in their late forties and it declined when the women were older than their late forties (Table 2; Fig. 2). The EC incidence peaked when the women were in their fifties (Table 2; Fig. 2).

**Table 1:** Characteristics of the women with endometrial hyperplasia or endometrial cancer from 2009 to 2012.

	2009	2010	2011	2012	Total	*p* value	Mean age (years)
Number of patients	598,917	614,451	630,083	633,973	2,477,424		
Numbers of patients with each disease							
Non-atypical EH	314 (21.4)	368 (25.1)	324 (22.1)	461 (31.4)	1,467 (100)	0.33^a^	44.1 ± 0.5^b^
Atypical EH	105 (26.1)	101 (25.1)	102 (25.3)	95 (23.6)	403 (100)	0.83^a^	44.1 ± 1.0^b^
EH	419 (22.4)	468 (25.1)^c^	425 (22.8)^c^	556 (29.8)	1,868 (100)	0.49^a^	44.1 ± 0.4^b^
Endometrial cancer	192 (22.1)	228 (26.3)	210 (24.2)	238 (27.4)	868 (100)	0.33^a^	52.7 ± 0.6^b^
Incidence per 100,000 woman-years^d^							
Non-atypical EH	28 (22–33)	33 (27–39)	27 (22–33)	33 (27–39)	30 (27–33)		
Atypical EH	6 (4–8)	6 (4–9)	8 (5–10)	6 (4–9)	7 (5–8)		
EH	34 (28–40)	39 (33–46)	35 (29–41)	39 (33–46)	37 (34–40)		
Endometrial cancer	7 (5–8)	8 (7–9)	7 (6–8)	8 (7–9)	7 (7–8)		

**Notes.**

The data are presented as the numbers (%), the numbers (95% confidence intervals), or the means ± standard deviations.

EH endometrial hyperplasia

a A weighted Pearson’s chi-square test was used to analyze the categorical data according to the year.

b The mean age was calculated using a weighted analysis method.

c One case had non-atypical endometrial hyperplasia and atypical endometrial hyperplasia simultaneously.

d The incidence rates were calculated using a weighted analysis method.

**Table 2:** The endometrial hyperplasia and endometrial cancer incidence rates from 2009 to 2012 stratified according to 5-year age increments.

Age (years)	Incidence per 100,000 woman-years (95% confidence interval)							
	Non-atypical EH		Atypical EH		EH		Endometrial cancer	
<15	0	0	0	0	0	0.0	0	0
16–20	4	(−1–8)	0	0	4	(−1–8)	0	0
21–25	11	(3–18)	2	(−1–6)	13	(4–22)	1	(0–1)
26–30	22	(12–32)	4	(0–8)	26	(15–37)	2	(1–3)
31–35	30	(20–41)	11	(4–17)	41	(28–54)	6	(3–9)
36–40	39	(27–51)	11	(5–17)	50	(36–63)	7	(4–9)
41–45	74	(59–89)	15	(8–21)	89	(72–105)	7	(5–8)
46–50	102	(84–120)	19	(13–26)	121	(102–141)	12	(9–14)
51–55	61	(47–74)	9	(5–13)	69	(55–84)	20	(16–24)
56–60	19	(11–27)	8	(2–15)	27	(17–37)	20	(17–23)
61–65	8	(3–13)	2	(1–3)	9	(4–14)	15	(12–18)
66–70	7	(2–12)	1	(0–2)	8	(3–13)	12	(9–15)
71–75	7	(1–13)	4	(−2–10)	11	(3–19)	9	(6–12)
>76	3	(0–7)	1	(0–2)	4	(0–8)	7	(3–11)
Total	30	(27–33)	7	(5–8)	37	(34–40)	7	(7–8)

**Notes.**

EH endometrial hyperplasia

## Discussion

Our study’s findings show that the EH and EC incidence rates were 37 per 100,000 woman-years (non-atypical EH: 30 per 100,000 woman-years; atypical EH: 7 per 100,000 woman-years) and 7 per 100,000 woman-years, respectively, and were somewhat lower than those determined from earlier studies. Previous studies that used data generated from 1985 to 2003, determined that the EH and EC incidence rates were between 133 and 208 per 100,000 woman-years (non-atypical EH: 121 per 100,000 woman-years; atypical EH: 17 per 100,000 woman-years) and 78 per 100,000 woman-years, respectively (Reed et al., 2009; Lacey et al., 2012). While the reasons for these differences are unknown, the times at which the studies were performed might have affected the incidence rates. Previous studies were based on data generated before 2002 when MHT was actively administered, but our study analyzed data that were generated after the WHI study (Rossouw et al., 2002; Haas et al., 2004; Anderson et al., 2004; Guay et al., 2007). The use of MHT has gradually declined since the WHI study’s findings provided evidence for the risks associated with MHT, and this may have affected the EH incidence. The incidence of EH among women in their thirties in the present study (41–50 per 100,000 woman-years) is similar to that reported by Reed et al. (2009) (23–63 per 100,000 woman-years). However, the ratio of the perimenopausal incidence of EH among women aged 45–54 years to the incidence of EH among women in their thirties was lower in our study (2.1) than that reported by Reed et al. (2009) (7.6). The ratio of the EH incidence among women aged around 60 years to the EH incidence among women who were in their thirties in the current study (0.4) showed a more distinct difference compared with that reported by Reed et al. (2009) (6.1). The mean menopausal ages are 51 years and 49 years in the United States and South Korea, respectively, and the EH incidence after perimenopause reported by Reed et al. was higher than that determined in the current study (Kato et al., 1998; Reed et al., 2009; Cho et al., 2014). Differences in the use of MHT may explain the disparities among the ratios, but the raw data analyzed in the studies differed and they were not adjusted for MHT usage. Unlike unopposed estrogen, estrogen and progestogen in combination do not increase the EH incidence (Furness et al., 2012), indicating that further studies are needed. Furthermore, like breast cancer, the age-related EH or EC rates might vary among countries. While breast cancer rates increase among women until they are in their late forties or early fifties in countries in the Far East, which include South Korea, Japan, and Taiwan, breast cancer rates in westernized countries increase consistently among women aged over 50 years (MacMahon, 2006; Lee et al., 2007). Obesity, which is a risk factor for EH and EC (Epplein et al., 2008; Carlson et al., 2012; Armstrong et al., 2012), may be another factor that underlies the disparities between the studies’ data. Unfortunately, our study and previous studies did not include the patients’ weights or body mass indices; hence, we were unable to analyze associations between the EH and EC incidence rates and obesity, or to compare our study’s data with those from previous studies. Further studies that adjust for obesity are needed.

Our study has several limitations. First, the data used in this study were based on diagnosis codes and we did not analyze the histological data. Hence, the EH and EC diagnoses were confirmed indirectly, and some EH or EC cases may have been wrongly diagnosed because of erroneous coding. However, patients with EH or EC were only selected when histological examinations had been carried out, so few wrongly diagnosed cases were analyzed in this study. Second, direct comparisons between our study and previous studies are limited, because the current study did not adjust for race, the body mass index, and MHT. The significance of this study’s data is that they verify the EH and EC incidence rates since 2009 and subsequent to the WHI study.

## Conclusion

In conclusion, the EH and EC incidence rates in South Korea from 2009 to 2012 were 37 per 100,000 woman-years and 8 per 100,000 woman-years, respectively. The EH and EC diagnosis frequencies peaked when the women were in their late forties and fifties, respectively. The EH and EC rates were somewhat lower in our study than those reported previously. Further studies are needed that adjust for race, MHT, and obesity.

## Additional Information and Declarations

### Data Availability

The following information was supplied regarding data availability: Yuk, Jin-Sung (2016): EH_incidence_2009_2012.sav.figshare.
https://dx.doi.org/10.6084/m9.figshare.3124015.v1.
